# Iron Status Predicts Malaria Risk in Malawian Preschool Children

**DOI:** 10.1371/journal.pone.0042670

**Published:** 2012-08-16

**Authors:** Femkje A. M. Jonker, Job C. J. Calis, Michael Boele van Hensbroek, Kamija Phiri, Ronald B. Geskus, Bernard J. Brabin, Tjalling Leenstra

**Affiliations:** 1 Global Child Health Group, Emma Children's Hospital, Academic Medical Centre, Amsterdam, The Netherlands; 2 Community Health Department, College of Medicine, Blantyre, Malawi; 3 Department of Clinical Epidemiology, Biostatistics and Bioinformatics, Academic Medical Centre, Amsterdam, The Netherlands; 4 Liverpool School of Tropical Medicine, University of Liverpool, Liverpool, United Kingdom; 5 Division of Internal Medicine, Centre for Tropical Medicine and Travel Medicine, Department of Infectious Diseases, Academic Medical Centre, University of Amsterdam, Amsterdam, The Netherlands; 6 Netherlands Ministry of Defence, The Hague, The Netherlands; University of Massachusetts Medical School, United States of America

## Abstract

**Introduction:**

Iron deficiency is highly prevalent in pre-school children in developing countries and an important health problem in sub-Saharan Africa. A debate exists on the possible protective effect of iron deficiency against malaria and other infections; yet consensus is lacking due to limited data. Recent studies have focused on the risks of iron supplementation but the effect of an individual's iron status on malaria risk remains unclear. Studies of iron status in areas with a high burden of infections often are exposed to bias. The aim of this study was to assess the predictive value of baseline iron status for malaria risk explicitly taking potential biases into account.

**Methods and materials:**

We prospectively assessed the relationship between baseline iron deficiency (serum ferritin <30 µg/L) and malaria risk in a cohort of 727 Malawian preschool children during a year of follow-up. Data were analyzed using marginal structural Cox regression models and confounders were selected using causal graph theory. Sensitivity of results to bias resulting from misclassification of iron status by concurrent inflammation and to bias from unmeasured confounding were assessed using modern causal inference methods.

**Results and Conclusions:**

The overall incidence of malaria parasitemia and clinical malaria was 1.9 (95% CI 1.8–2.0) and 0.7 (95% CI 0.6–0.8) events per person-year, respectively. Children with iron deficiency at baseline had a lower incidence of malaria parasitemia and clinical malaria during a year of follow-up; adjusted hazard ratio's 0.55 (95%-CI:0.41–0.74) and 0.49 (95%-CI:0.33–0.73), respectively. Our results suggest that iron deficiency protects against malaria parasitemia and clinical malaria in young children. Therefore the clinical importance of treating iron deficiency in a pre-school child should be weighed carefully against potential harms. In malaria endemic areas treatment of iron deficiency in children requires sustained prevention of malaria.

## Introduction

Childhood iron deficiency is prevalent globally with highest estimates in children in developing countries. In sub-Saharan Africa, 33–66% of children are iron deficient, most of whom are under the age of five years [1], [2]. The high prevalence of iron deficiency in these populations is a consequence of low nutritional iron intake due to low economic status [2], decreased absorption of dietary iron due to a high prevalence of infections [3] or intestinal blood loss in the case of helminth infections [4]. Iron deficiency causes anemia and developmental dysfunction [2], [3] and the current consensus is to treat all iron deficient children [5]. Conversely, evidence of a protective effect of iron deficiency against malaria and other infections exists [6]–[12].

In recent years concerns have focused mainly on the possible harmful effects of treating iron deficiency rather than on the effects of iron status itself [13]–[19]. A recent Cochrane meta-analysis reviewing iron supplementation in children in malaria endemic areas concluded that iron supplementation did not adversely effect children in malaria endemic areas when adequate malaria surveillance is provided, although the authors considered their data were limited by various factors including lack of data on baseline iron status [20]. There is little conclusive data on the protective effect of iron deficiency against malaria. The reliability of current data on iron status is limited since available iron biomarkers are affected by inflammation which makes interpretation of results from malaria endemic areas problematic [21]. In addition, the complexity of factors influencing both malaria susceptibility and iron status are not always taken into account, potentially leading to confounded results. To improve control and management of malaria as well of iron deficiency, reliable data about the influence of iron status on malaria risk are needed. The aim of this study was to assess the predictive value of baseline iron status in children for subsequent malaria risk, explicitly taking misclassification of iron deficiency due to concurrent inflammation and confounding bias into account by utilizing modern causal inference methods in the analyses of a dataset of Malawian pre-school children.

## Methods

### Study Design

This is a secondary analysis of follow-up data from a cohort of pre-school children recruited as the control group for the *Severe Anemia Study* (*SevAna study*), a large case-control study investigating the etiology, pathophysiology and outcome of severe anemia in southern Malawi [22]–[24]. The analysis is restricted to the non-severely anemic control population (hemoglobin level ≥5.0 g/dL), because the cases all received a blood transfusion. Follow-up data of the first year after enrolment were used, allowing the observation of a full year of malaria transmission per child.

### Ethical Statement

The *SevAna study* was approved by the Ethics Committees of the College of Medicine, Malawi (ref. no P.00/01/116, and the Liverpool School of Tropical Medicine, United Kingdom (ref. no 01.29). Prior to enrolment in the study, written informed consent was obtained from the parent or guardian of all study participants.

### Study population

The *SevAna study* recruited 381 cases and 757 controls between July 2002 and July 2004 (Figure S1). Every child (6–60 months old) presenting at the hospital with severe anemia (hemoglobin <5.0 g/dL) was defined as a case. For each case, two controls were enrolled; a community control living within 100–1000 meter of the case, and a hospital control, which was the first child presenting at the outpatient department on the day following presentation of the case patient. Controls were eligible for recruitment if aged 6–60 months and if their hemoglobin level was at least 5.0 g/dL. All children were visited at 1, 3, 6 and 12 months from date of recruitment (active follow-up). If children did not report for follow up, families were visited by the study team. In addition, guardians were asked to present their child to a study-clinic whenever the child was sick (passive follow-up).

### Study setting

The *SevAna study* took place in Malawi, a land-locked country in the south-eastern part of Africa of which 20% is covered by Lake Malawi. The climate is tropical, but prevalence of malaria and other infectious diseases vary with proximity to the lake and altitude. Rough estimates of the prevalence of iron deficiency vary from 20–40% [2], [25]. Study participants were recruited in two settings; Blantyre district (study site: Queen Elizabeth Central Hospital) and Chikwawa District (study site: Chikwawa District Hospital), both in the southern region of Malawi. Blantyre is the main commercial town of Malawi with a predominantly urban population of half a million. At an altitude of 800 m above sea level malaria is mainly seasonal (approximately 1 infectious bite per person per year) [26]. Chikwawa District Hospital, which caters for a predominantly rural population of approximately 400 thousand people, is situated in the lower Sire Valley, 50 km south of Blantyre. With an altitude of 250 m above sea level malaria transmission is year round (approximately 170 infectious bites per person per year) (Milahowa T, personal communication).

### Clinical procedures

At recruitment a detailed medical and socio-economic history was recorded and a physical examination was performed. Samples of blood, stool and urine were collected. Children were treated if indicated using local treatment guidelines. Clinical malaria was defined as a positive blood slide with concurrent fever (axillary temp >37.5°C) or history of fever (caregiver recall of fever in the last week). Severe malaria was defined as a positive blood slide with either severe anemia (hemoglobin <5.0 g/dl) or coma [27]. Malaria was treated with sulfadoxine-pyrimethamine (SP) and if the child was unable to take oral medication parenteral quinine was administered. As a standard procedure at recruitment all study participants received presumptive malaria treatment (25.0/1.25 mg/kg SP) and according to local guidelines all children received iron supplementation 2 mg/kg/day for 28 days. Follow-up procedures existed of a medical history, physical examination and a blood sample to determine hemoglobin and malaria parasitemia, and if indicated children were treated using local treatment guidelines. Deaths were recorded and if they occurred outside the study clinics they were investigated as completely as possible using a verbal autopsy form.

### Laboratory procedures

Hemoglobin (Hb) concentrations were measured by HemoCue B-Hemoglobin analyzer (HemoCue, Ängelholm, Sweden) to judge eligibility-criteria. Hb measured by Coulter counter analyzer (Beckman Coulter, Durban, South Africa) was used for statistical analyses. Moderate and severe anemia were defined as an Hb of <11.0 g/dL and <5.0 g/dL, respectively. Plasma levels of C-reactive protein (CRP) and serum ferritin were analyzed on Modular P800 and Monular Analytic E170 systems (Roche Diagnostics, Switzerland). Iron deficiency was defined as serum ferritin <30 µg/L [25]. Inflammation was defined as CRP>10 g/L. For malaria diagnoses thin-film blood slides were read by two independent readers; with a third reader being used if results differed by 25% or more. The number of *Plasmodium falciparum* asexual parasites per 200 white cells was counted and expressed as the number per microliter of blood. Malaria parasitemia was defined as the presence of one or more *Pl. falciparum* asexual parasites [28]. Human immunodeficiency virus (HIV) infection was assessed using two rapid tests (Determine, Abbott Laboratories; and Uni-Gold, Trinity Biotech). Discordant or reactive rapid-test results in children less than 18 months of age were resolved by PCR [29].

### Statistical Analyses

The incidence of clinical malaria and parasitemia was calculated for a follow-up period of one year. Multiple events per child were registered. A child was considered not to be at risk for incident malaria for the 28 days following malaria treatment (SP), as the prophylactic effect of malaria treatment was assumed [30]. This 28 day period was excluded as person-time and malaria events during this period were not counted for analyses. Malaria parasitemia or clinical malaria occurring during this period were defined as treatment-failure and were excluded from analysis.

**Figure 1:**
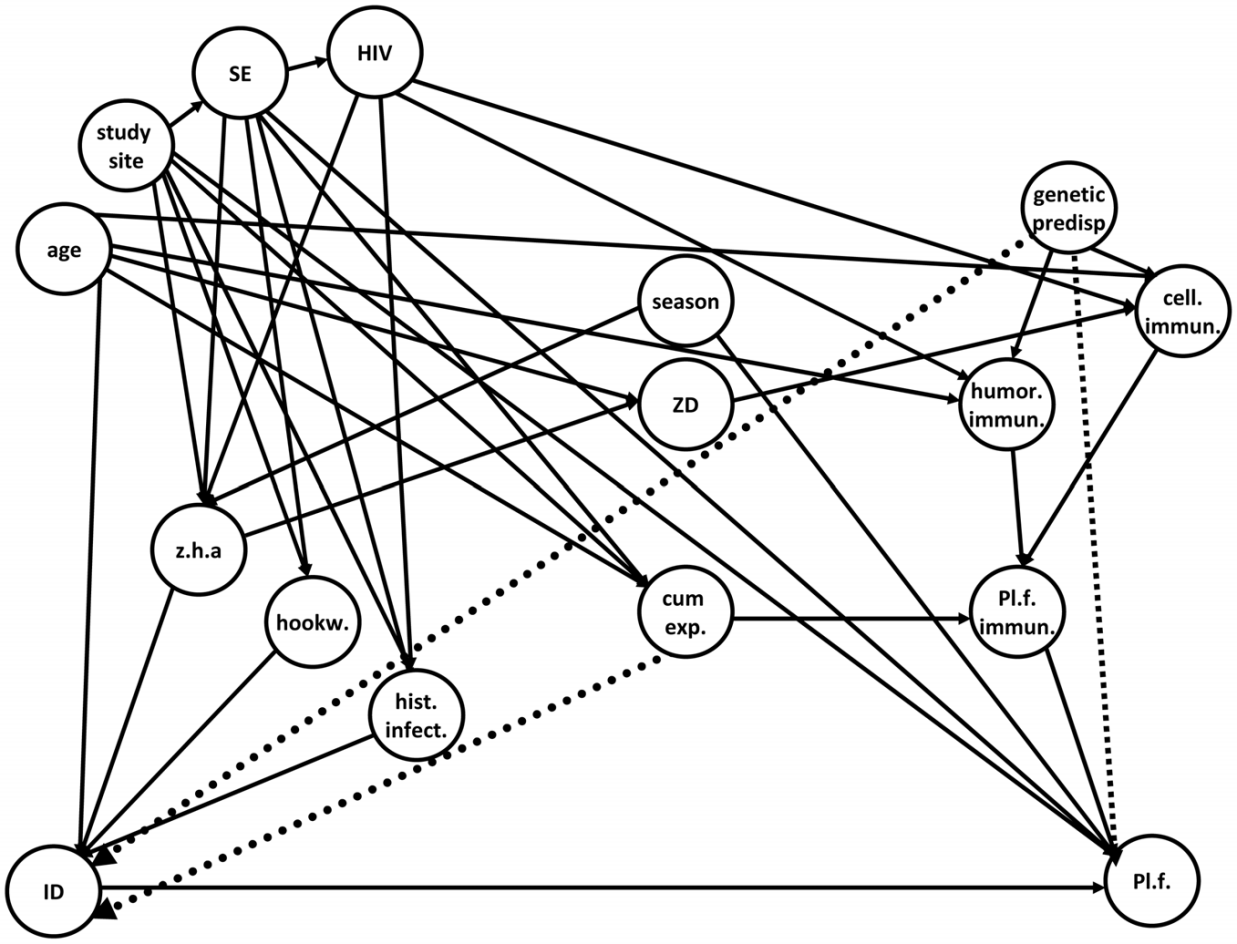
Directed acyclic graph for the relation between iron deficiency and malaria risk. cell. immun: cellular immunity; cum exp: cumulative malaria exposure; genetic predisp: genetic predisposition; humor immun: humoral immunity; hookw: hookworm infection; hist infect: history of other infections than malaria; HIV: human immunodeficiency virus; ID: iron deficiency; Pl.f.immun: immunity for Pl.falciparum; Pl.f: Plasmodium falciparum infection; SE: socio economic score; ZD: zinc deficiency; z.h.a: z-score height for age.

The main predictor, iron deficiency, was defined as serum ferritin <30 µg/L. Possible confounders considered for inclusion were: sex, age, area of residence, season, socio economic status, genetic predisposition of malaria, cellular immunity, humoral immunity, cumulative malaria exposure, malaria immunity, history of other infections than malaria, HIV infection, hookworm infection, nutritional status and zinc deficiency (figure 1). We used DAGitty®, a web-based application based on the *Directed Acyclic Graph theory*, to model the causal relationship between iron status, malaria risk and any potential (measured and unmeasured) confounders [31], [32]. This DAG-program was used to identify sets of confounders that together fully adjust for confounding in multivariable modeling [31], [33], [34]. Several of these sets of confounders *(minimally sufficient adjustment sets)* were identified from figure 1. The set containing HIV-status, socio economic status, age, nutritional status and study site was used for analysis, as these variables were available in our data set. Socio economic status was scored on parents' education (1–4), job (1–4) and number of assets (0–6). Chronic malnutrition defined as height-for-age <−2 SD was used as the marker for nutritional status [35].

Due to inadequate sample volume in 29% of the children serum ferritin was not available. The missing data were assumed to be missing at random; basic characteristics between children with and without available ferritin assessments were comparable (Table S1). Because complete-cases analysis with a large amount of missing values may lead to loss of efficiency and bias [36], missing data were imputed using multiple imputation by chained equations in the R software version 2.14.0 utilizing the MICE package [37], [38]. The imputation model included all baseline covariates in table 1 plus soluble transferrin receptor (sTfR) and mean corpuscular hemoglobin concentration (MCHC) as additional markers of iron status. The outcome variable was included in the imputation model via the event indicator and the Nelson-Aalen estimator of baseline hazard, as studies suggest that these specific components lead to lower bias in estimation [39]. Regression estimates and their standard errors from analyses of the 25 separate imputed data sets were summarized using Rubin's rules [40].

**Table 1:** Baseline characteristics of the study population.

Characteristic	Result
Female	357/727 (49.1%)
Age in months: mean (s.d)	23.9 (12.6)
Age<2 years	408/727 (56.1%)
Living in an urban area	376/727 (51.7%)
SE-score: mean (s.d)	7.2 (2.1)
Hospital control	357/727 (49.1%)
Hemoglobin: mean (s.d)	9.7 (2.1)
Iron deficiency	146/513 (28.5%)
Malnourished	275/669 (41.1%)
Fever (>37.5°C axillary)	205/727 (28.4%)
CRP>10 mg/L	373/630 (59.2%)
Malaria parasitemia	308/720 (42.8%)
Clinical malaria	108/720 (14.9%)
HIV-infected	39/670 (5.8%)

Hemoglobin in g/dl; CRP: C-Reactive Protein; malnourished defined as height-for-age <−2 SD. HIV: Human Immunodeficiency Virus. Iron deficiency defined as serum ferritin <30 ug/L. SE-score: Socio Economic Score, a sum of the following scores: parents' education (1–4), job parents (1–4) and number of assets (0–6).

To compare the incidence of malaria during follow-up between iron deficient and iron replete individuals, a marginal structural Cox proportional hazards model was used to adjust for confounding. Marginal structural models are a new class of statistical models to estimate causal effects [41], [42], and are equivalent to non-parametric multivariate standardization methods [41], [43], [44]. Effect estimates can be adjusted for measured confounders using inverse probability of exposure weighting. In addition, marginal structural models allow the quantitative evaluation of unmeasured confounding [42], [45], [46]. Stabilized inverse probability weights were estimated using the ‘ipw’ package in R [30], [47]. To adjust for the correlation between recurrent malaria events in a single person, robust standard errors were computed. All covariates were treated as time-independent. The proportional hazards assumption was checked by plotting the scaled *Schoenfeld* residuals against time and tested using cox.zph procedure in R [48]. We assessed whether the effect of iron deficiency on malaria was modified by age or study site by testing their interaction terms with the Wald statistic. Interaction terms were considered significant if p<0.1.

We assessed to which extent the outcomes could have been biased because of possible misclassification of the iron deficiency indicator (serum ferritin), by reanalyzing the data using a more sensitive and a more specific definition of iron deficiency [49]; cut-offs for serum ferritin that varied by inflammation status; children without inflammation (CRP<10 g/L) were defined as iron deficient having a serum ferritin of <12 µg/L; for children with current inflammation (CRP>10 g/L) serum ferritin cut-offs of 30 and 70 µg/L were explored [25], [50].

In addition, to evaluate how large the average difference in susceptibility to malaria at baseline between the iron deficient and iron-replete group would need to have been to partly or fully explain our observed differences in malaria incidence, we used a method for sensitivity analysis for unmeasured confounding developed by *Robins et al.* [42], [45], [46]. The method requires that unmeasured confounding is quantified as a bias parameter u = α^(2×ID - 1)^, with ID = 1 or 0 as indicator of iron deficiency and α the degree of unmeasured confounding or, in other words, the average difference in malaria risk at baseline on a ratio-scale and conditional on the covariate distribution.

## Results

### Study population

Follow-up data were available for 96.0% of all study participants (727/757). Baseline characteristics are presented in table 1. Baseline characteristics of children with and without follow up data were comparable (data not shown). Of the remaining 727 children the attendance at active follow-up visits ranged from 86–89%.

### Incidence of malaria parasitemia and clinical malaria

During the follow-up year the overall incidence of malaria parasitemia and clinical malaria was 1.9 (95% CI 1.8–2.0) and 0.7 (95% CI 0.6–0.8), respectively. This incidence was higher in the rural areas and in the children less than 24 months of age (Table 2). There were four cases of severe malaria, three of which were iron replete. During follow-up 0.7% (1/141) of the iron deficient children and 1.3% (5/372) of iron replete children died.

**Table 2:** Incidence of malaria parasitemia and clinical malaria per person year.

		All		Iron deficient group		Iron replete group	
		Events	Incidence	Events	Incidence	Events	Incidence
Malaria parasitemia	All	896	1,9	180	1,3	716	2,2
	Urban	253	1,0	38	0,5	215	1,1
	Rural	643	3,1	142	2,2	501	3,6
	Age<24 months	557	2,1	139	1,4	418	2,5
	Age≥24 months	339	1,6	41	0,9	298	1,8
Clinical malaria	All	318	0,7	58	0,4	260	0,8
	Urban	65	0,2	11	0,1	54	0,3
	Rural	253	1,2	47	0,7	206	1,5
	Age<24 months	207	0,8	44	0,5	163	1,0
	Age≥24 months	111	0,5	14	0,3	97	0,6

Incidence of malaria parasitemia and clinical malaria per person year stratified per area, age group and iron status. Malaria parasitemia defined as a positive malaria blood slide; Clinical malaria: positive malaria blood slide with concurrent fever (axillary temp >37.5°C) or history of fever.

### Baseline iron status and incidence of malaria

The marginal structural Cox model showed that iron deficiency was an independent predictor of malaria parasitemia and clinical malaria; HR (95%CI) 0.55 (0.41–0.74) and 0.49 (0.33–0.73) respectively (Table 3). The effect of iron deficiency on malaria parasitemia and clinical malaria was consistent across different sub-populations; no significant effect measure modification by study site (urban vs. rural) or age (linear term) was seen (all p-values>0.9). In addition, the effects were sustained throughout the study period; the proportional hazards assumption was not refuted and when analysis was done on consecutive periods of 4 months, consistent effect measures were seen (data not shown). Analyses conducted with the complete cases data set (including only children with a ferritin result) showed comparable results (HR (95%CI) 0.61 (0.44–0.81) and HR (95%CI) 0.53 (0.36–0.78) for malaria parasitemia and clinical malaria respectively.

**Table 3:** Hazard ratios (95% Cl) of iron deficiency for the risk on malaria parasitemia en clinical malaria.

	univariate Cox model		marginal-structural Cox model	
	Malaria parasitemia	Clinical malaria	Malaria parasitemia	Clinical malaria
Definition iron deficiency	HR (95% CI)	HR (95% CI)	HR (95% CI)	HR (95% CI)
SF<30	0.59 (0.44–0.78)	0.54 (0.36–0.80)	0.55 (0.41–0.74)	0.49 (0.33–0.73)
SF<30(CRP>10)/SF<12(CRP<10)	0.57 (0.40–0.82)	0.53 (0.32–0.89)	0.46 (0.31–0.71)	0.43 (0.25–0.73)
SF<70(CRP>10)/SF<12(CRP<10)	0.66 (0.52–0.85)	0.59 (0.41–0.84)	0.60 (0.46–0.79)	0.52 (0.35–0.75)

Iron deficiency is defined with different cut-offs for serum ferritin (SF) in ug/L, depending on presence of inflammation, defined as C-reactive protein (CRP) >10 mg/L. The marginal-structural Cox model included HIV-infection, socio economic score, age, nutrition and study site.

### Sensitivity analyses

**Figure 2:**
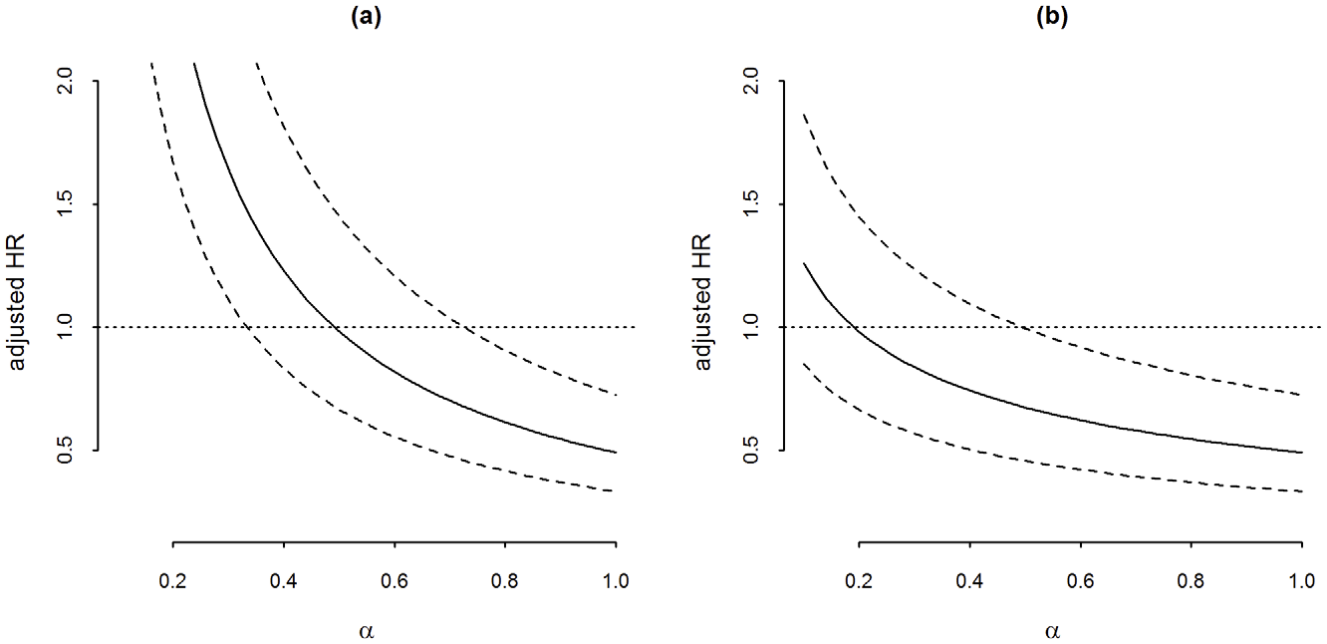
Sensitivity analysis of the impact of unmeasured confounding on the effect of baseline iron deficiency on risk of clinical malaria. The y-axis represents the hazard ratio for malaria infection comparing iron deficiency to iron replete after adjustment for average differences in prior risk represented by α on the x-axis. Figure 2a represents analyses in which net amount of confounding is specified for both iron status groups (bias parameter u = α^(2×ID - 1)^). Figure 2b represents analyses in which the amount of confounding was varied as a function of age (bias parameter u = α^(2×ID - 1)×(1 - abs(age-36)/30^).

Analyses assessing sensitivity to misclassification of iron status are presented in Table 3 and show consistent results using different definitions of iron deficiency. Hazard ratio estimates for clinical malaria from the marginal structural Cox model including adjustment for varying amounts of potential unmeasured confounding are presented in Figure 2. Two distinct distributions of unmeasured confounding were considered. Figure 2a presents results from analyses in which the net amount of bias per group is specified. The a priori clinical malaria risk in the iron deficient group should have been at least 0.72 times the a priori risk in the iron replete group to remove significance of our findings, and 0.48 times to fully explain our findings (Figure 2a). Figure 2b presents results from analyses in which the amount of unmeasured confounding varied as function of age; increasing log-linearly up to a peak at the age of 3 years and then decreasing to no confounding effect. The ratio of priori risks would have to have been at least 0.49 at its peak at 3 years-of-age to remove significance and 0.18 to fully explain our finding (Figure 2b). Baseline malaria is often included as a proximate measure of susceptibility to malaria in multivariable analyses. However, since our hypothesis was that baseline iron status influences malaria risk, baseline malaria would technically not be a confounder, i.e. the exposure may not affect the confounder (Causal knowledge as a prerequisite for confounding evaluation [33]). We therefore chose to assess the effect of differences in malaria susceptibility at baseline through sensitivity analysis. Notwithstanding, when baseline malaria parasitemia respectively clinical malaria was included, effect estimates were slightly attenuated though still significant HR (95%CI) 0.66 (0.48–0.91) and HR (95%CI) 0.51 (0.33–0.79) for malaria parasitemia and clinical malaria respectively.

## Discussion

In this study, children with iron deficiency at baseline (serum ferritin <30 µg/L) had a lower incidence of malaria parasitemia and clinical malaria during a follow up period of one year. This finding suggests that iron deficiency protects against malaria parasitemia and clinical malaria in pre-school children living in malaria endemic areas. The results were consistent across study sites with varying transmission intensity and across age-groups despite changing susceptibility with age. In addition, the difference in risk between baseline iron status groups persisted throughout the follow-up period.

Our findings are in line with results of the few other studies assessing influence of iron status on malaria risk [10], [51]. The protective effects of iron deficiency on malaria risk shown in these two studies, were of similar magnitude, yet Gwamaka *et al* also showed an effect on all cause and malaria associated mortality [51].

As with any observational study, the results of this study are subject to bias. Therefore, we explicitly evaluated the potential impact that misclassification and confounding bias could have had on our results. Firstly, because serum ferritin levels are influenced by concurrent inflammation it may not adequately reflect true iron status. As a consequence, misclassification of baseline iron status may have occurred and thus resulted in biased effect measures. We evaluated whether use of a more sensitive and a more specific definition, taking concurrent inflammation into account, would substantially change the results. As expected, use of a more specific definition slightly increased the magnitude of effect and the use of a more sensitive definition led to a slightly weaker effect. Importantly, this did not change our conclusions, suggesting that the findings are robust to misclassification bias.

In the directed acyclic graph (DAG) in Figure 1 all potential confounders of the effect of iron status on malaria risk as conceptualized by the authors, were evaluated. We considered two additional yet unmeasured variables which could theoretically confound our results by both protecting against malaria and causing iron deficiency. We assessed how strong these potential confounding effects should have been to explain the observed difference in malaria risk between iron status groups. The first unmeasured factor was ‘cumulative exposure to malaria’. Recent studies have suggested that chronic infection, including malaria, may reduce iron absorption in the gut by inducing an increase of hepcidin [52]–[54] and that malaria infection may lead to iron loss through hemosiderin deposition. In addition, repeated malaria infections (cumulative exposure) result in development of immunity to malaria. The age at which clinical immunity is attained is influenced by local infection pressure represented by the number of infectious bites per year (EIR) [55]. If it is true that increased exposure to malaria simultaneously induces iron deficiency and increased malaria immunity, cumulative exposure is a potential confounder in our analyses. In other words, the lower incidence of malaria in the iron deficient group may have been due to higher malaria exposure and subsequently better immunity to malaria, rather than directly due to mechanisms involving iron status. We conducted sensitivity analyses allowing unmeasured confounding bias to vary as a function of age; reflecting the theoretical confounding effect of cumulative exposure, where the difference in immunity (and thus the maximum prior risk difference) gradually changes with increasing age, reaching a peak at the age of 36 months [56] to subsequently decrease after that. The average prior risk of malaria should then have been at least half the risk in the iron replete group at the peak age of 36 months to have led to a spurious significant finding (Figure 2b). Although theoretically a plausible confounder, it is unlikely that in reality ‘cumulative exposure to malaria’ would result in such large differences in prior malaria risk, suggesting that our measured effects are unlikely due to confounding by differences in cumulative exposure. As a second potential confounding factor we considered a (yet undiscovered) genotype simultaneously causing iron deficiency and increasing immunity to malaria, for example a variant in iron metabolism that inhibits maturation of *Plasmodium* in the red cells [57]. Our analysis showed that to be responsible for our statistically significant finding, the average prior risk of malaria in the iron deficient group should have been at least 0.72 times the risk compared to that of the iron replete group (Figure 2a). A genetic predisposition causing a 28% lower prior risk is imaginable (i.e. sickle cell trait) and if such a genotype is discovered this may change the interpretation of our results.

In the interpretation of our analyses we assume that iron status measured at baseline remained relatively stable throughout the year of follow-up. Unfortunately, iron status measurements were not repeated after baseline, so we are not able to check this assumption. It is possible that children changed from the iron deficient group to the iron replete group and vice versa. However, because our study population was closely followed and adequately treated for infections it is more likely that iron deficient children became iron replete than the other way around. The resulting misclassification would have resulted in an underestimation of the effect.

For the underlying mechanism of the protective effect of iron deficiency on susceptibility to malaria infection, several hypotheses have been raised. It is suggested that iron deficiency alters the membrane structure of parasitized erythrocytes which facilitates their elimination by phagocytic cells, yet further research is needed to further support this hypothesis [58]. Another hypothesis is that the increased zinc protoporphyrin in iron deficient parasitized red blood cells binds to heme crystals and thus inhibits the formation of hemozoin, in analogue to antimalarial quinolines [59].

We cannot fully exclude that the observed effect was the result of increased susceptibility in the iron replete group resulting from the month of iron supplementation, rather than a protective effect of iron deficiency. In 2006 a large supplementation trial in Zanzibar was stopped prematurely after increased morbidity and mortality was observed in iron replete children receiving iron and folic acid [19]. However it remains unclear whether the increased morbidity was due to the effect of the iron, or the folic acid component of the supplements [60], [61]. Although the month of iron supplementation in this study complicates the interpretation of our study results, it is a reflection of current practice in this such settings in which short courses if iron supplementation are routinely given. In addition we think its influence was minimal for several reasons. Firstly, since the difference in risk was still observable towards the end of follow-up, the influence of the one month of iron supplementation was likely to be negligible. Secondly 59% of the children had an infection at baseline and this was likely to inhibit the absorption of iron in the first two weeks of supplementation [52]–[54].

In conclusion, iron deficiency seems to protect against malaria parasitemia and clinical malaria. However, because we lack direct measures of baseline immunity to malaria our results should be confirmed by well conducted randomized controlled trials or observational birth cohort studies. Our findings support the theory of nutritional adaptation to infectious diseases [62], [63]. To avoid increased malaria risk due to “neutralizing" the protective effect of iron deficiency, treatment of iron deficiency should occur under simultaneous and sustained control of prevalent infections. We recommend that treatment of iron deficiency occurs within the context of the Integrated Management of Childhood Illness (IMCI) and will benefit from the addition of Integrated Vector Management (IVM) [64], [65]. However, because oral iron is often poorly absorbed in individuals with concurrent inflammation [52]–[54], prevention of iron deficiency may be more effective. Treatment and especially prevention of infections, in particular hookworm infections [4], [66], will contribute to prevention of iron deficiency through decreased loss and increased absorption of dietary iron. Whichever strategy is chosen, concurrent and sustained prevention of malaria through Integrated Management of Childhood Illness is imperative in malaria endemic areas.

## Supporting Information

Figure S1 **Flow chart study population.** This flowchart presents number of children enrolled in the main study, a case control study investigating etiology of severe anemia (I); number of children enrolled in the cohort study (II) and the number of children attending the follow-up visits (III).
(PDF)

Table S1 **Baseline characteristics of the study population.**
(DOC)
