# Supplementary figures and images for: Iron Status Predicts Malaria Risk in Malawian Preschool Children
Source: PLoS One. 2012 Aug 16;7(8):e42670. doi: 10.1371/journal.pone.0042670 (PMC3420896; doi:10.1371/journal.pone.0042670)

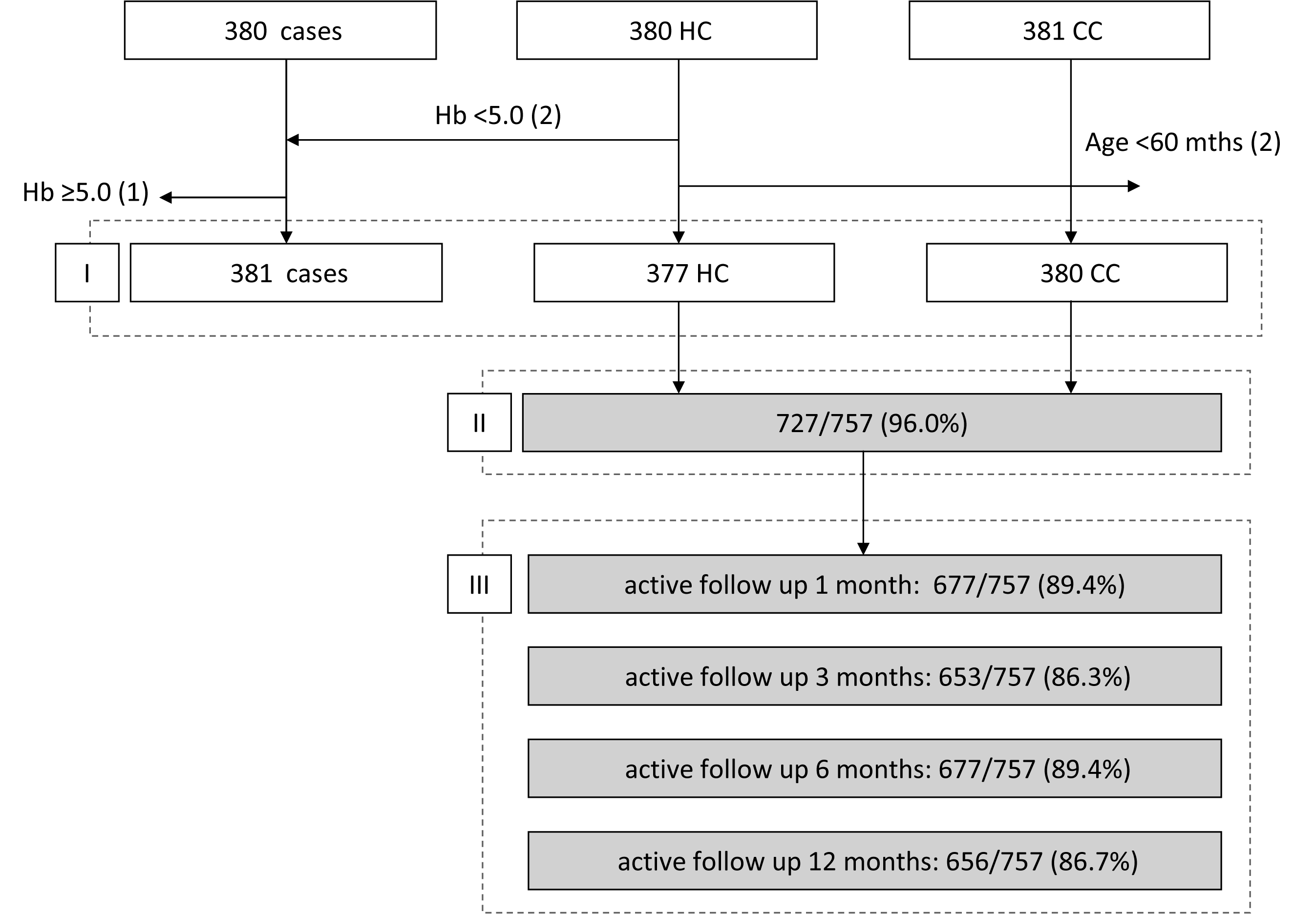

Supplement: Figure S1 — Flow chart study population. This flowchart presents number of children enrolled in the main study, a case control study investigating etiology of severe anemia (I); number of children enrolled in the cohort study (II) and the number of children attending the follow-up visits (III). (PDF) [file pone.0042670.s001.pdf]
