# Supplementary material for: Iron Status Predicts Malaria Risk in Malawian Preschool Children
Source: PLoS One. 2012 Aug 16;7(8):e42670. doi: 10.1371/journal.pone.0042670 (PMC3420896; doi:10.1371/journal.pone.0042670)
Supplement: Table S1 — Baseline characteristics of the study population. (DOC) [file pone.0042670.s002.doc]

| **Table S1. Baseline characteristics of the study population.** | | |  |
| --- | --- | --- | --- |
| **Characteristic** | **All** | **ferritin available** | **complete cases** |
| Female | 357/727 (49,1%) | 239/513 (46,6%) | 195/438 (44,5%) |
| Age in months: median (IQR) | 21,8 (13,4-32,7) | 23,3 (13,7-33,3) | 23,7 (14,2) |
| Age < 2 years | 408/727 (56,0%) | 268/513 (52,2%) | 223/438 (50,9%) |
| Living in an urban area | 376/727 (51,7%) | 264/513 (51,5%) | 196/738 (44,7) |
| SE-score: mean (s.d) | 7.2 (2.1) | 7.2 (2.1) | 7,2 (2,1) |
| Hospital control | 357/727 (49,1%) | 253/513 (49,3%) | 213/438 (48,6%) |
| Malnourished | 271/669 (40,5%) | 19/513 (42,0%) | 185/438 (42,2%) |
| Fever (> 37.5 °C axillary) | 205/727 (28,4%) | 142/513 (27,9%) | 119/438 (27,4%) |
| CRP > 10 mg/L | 374/630 (59.4%) | 283/513 (55,4%) | 238/438 (54,6%) |
| Malaria parasitemia | 308/720 (42,8%) | 207/513 (40,6%) | 180/435 (41,4%) |
| Clinical malaria | 108/720 (15,0%) | 71/513 (13,8) | 60/438 (13,7%) |
| HIV-infected | 39/670 (6,8%) | 30/513 (6,3%) | 38/438 (6,6%) |
| Hemoglobin: mean (s.d) | 9.7 (2.1) | 9,8 (2,0) | 9,8 (1,9) |
| Hemoglobin in g/dl; CRP: C-Reactive Protein; malnourished: height-for-age < -2 SD | | | |
| HIV: Human Immunodeficiency Virus. Iron deficient denied as serum ferritin < 30 ug/L. | | | |
| SE-score: Socio Economic Score, a sum of the following scores: parents’ education (1 - 4), | | | |
| job parents (1 - 4) and number of assets (0-6). | |  |  |
